# Professional Infamy: The Medical Council and the Obstetrical Society

**Published:** 1895-01-26

**Authors:** 


### The Hospital

An Institutional Journal of

The Medical Sciences and Hospital Administration, with

AN EXTRA NURSING SUPPLEMENT.

Vol. XVII.—No. 435.] [Jan. 26, 1895.

### Professional Infamy: The Medical Council and the Obstetrical Society.

The question of "Professional Infamy," always recognised to be one of special importance amongst members of the medical profession, has suddenly assumed proportions of quite startling magnitude. The General Medical Council has decided by resolution that all persons not authorised by statute to grant certificates of competency in midwifery shall be held guilty of "infamous conduct in a professional respect" if they grant such certificates after a date fixed by the Council. This is a resolution which lacks nothing in point of decidedness: does it lack anything in point of expediency or wise statesmanship?

In an ancient civilisation like our own there are always many institutions which have, so to speak, sprung naturally from the soil. They have arisen as occasion seemed to demand; they have attained to a certain growth and recognition; and the fact that they have been permitted to live for a considerable length of time gives them, as a rule, if not the right to live on indefinitely, at least the right to be permitted to say what they can in their own defence before being summarily decapitated. This we lay down as a comprehensive proposition, which covers a great variety of existing institutions, both medical and non-medical. Side by side with this we would place another proposition, namely, that it is seldom good policy to extinguish existing institutions suddenly, and more especially to do so without allowing them the opportunity of being heard in their own defence. Now the action of the General Medical Council peremptorily prohibits the issue of midwifery certificates by individual medical practitioners after a given date; and we must all agree that that is a thing well done, inasmuch as no self-respecting practitioner should ever have taken it upon himself to issue such certificates. With the suppression of such private and irresponsible certifiers, we repeat, all must agree. But the action of the Council also suppresses such public and responsible bodies as the Obstetrical Society of London, and with the same undiscriminating peremptoriness. This, we venture to suggest, is a question of a different complexion altogether.

Does the General Medical Council intend to put its foot down against the training and certification of midwives in permanence? So far as appears it does not. But even if it did, that would be no justification for what cannot but be regarded by persons trained to political thought and action as a piece of unfriendly, unjust, hasty, and high-handed legislation. Let it be granted that the Obstetrical Society ought to be disestablished in the interests of a new statutory provision of midwives for the poorer portion of the public. Is that a reason why a society with an honourable record of good and useful work should be not merely flouted and treated without consideration of any kind, but declared retrospectively and prospectively guilty of professional infamy if it dares to make a single claim in practice on behalf of its own continued existence and work as a teacher and certifier of midwives? It is but too obvious that there has been no statesmanship at all present at the December meetings of the General Medical Council. Raw haste, ill considered conclusions, an unmannerly disregard alike for established institutions and for high professional reputations, these have marked the December deliberations of the Council, and have branded every member of the voting majority with the unmistakable sign, if not of "professional infamy," of political incompetency.

Sir John Williams, in a dignified protest, has pointed out some of the consequences which flow naturally from the decision of the Council. "I can hardly think," says Sir John, "that the Council can be fully aware of the full significance of its resolution, for should the society," that is the Obstetrical Society, "grant its certificate again, the following persons will be regarded as guilty of 'infamous conduct,' by the General Medical Council, namely all the past presidents of the society, its trustees, and officers, most of the examiners in midwifery in the Universities of London, Oxford, and Cambridge, and the Royal Colleges of Physicians and Surgeons. In fact, all the obstetric physicians of note in London. These men, contingently branded with 'infamy,' are well acquainted with the Medical Acts, and many, or all of them, well versed in the ethics of the profession and punctilious in their observance, many of them having held high posts in the colleges and schools, and are held in honour by their professional brethren; none are better informed of the needs of the poor in their travail, none are better able to train and examine midwives, and, by reason of their learning, culture, and experience, none are fitter to form correct views of what is and what is not professionally ' infamous.' "

Now every sober-minded medical man who carefully and deliberately studies this protest of Sir John Williams will perceive with assured conviction that Sir John is in the right, and that the General Medical Council has put itself altogether in the wrong. Of course it is open to the Council to rescind its resolution, and it must do so. "Professional Infamy" is not a mere phrase to be tossed about from mouth to mouth and placed jauntily on the medical statute book in the irresponsible half-hour which follows a light-hearted midday luncheon. It is a tremendous fact; a fact which covers a man not merely with "professional" but with real "infamy" when justice and sobriety have pronounced the fatal verdict. It is the fate of almost all persons, public bodies as well as private individuals, sometimes to go wrong. Happy is that body which, having rashly stepped out of the way of plain sense and judicious conduct, has the courage and rightmindedness to promptly undo its own acts.

